# Neoirietriol

**DOI:** 10.1107/S1600536810022336

**Published:** 2010-06-26

**Authors:** Hiroki Takahashi, Yoshinori Takahashi, Minoru Suzuki, Tsuyoshi Abe, Michio Masuda

**Affiliations:** a Graduate School of Human and Environmental Studies, Kyoto University, Kyoto 606-8501, Japan; b Graduate School of Environmental Earth Science, Hokkaido University, Sapporo 060-0810, Japan; c The Hokkaido University Museum, Sapporo 060-0810, Japan; d Division of Biological Sciences, Graduate School of Science, Hokkaido University, Sapporo 060-0810, Japan

## Abstract

The title compound {systematic name: (1*R*,4*S*,4a*S*,7*R*,8a*R*)-4-bromo-7-[(1*S*,3*R*)-3-bromo-1,2,2-trimethyl­cyclo­pent­yl]-1,4a-dimethyl­deca­hydro­naphthalene-1,7,8a-triol}, C_20_H_34_Br_2_O_3_, is a neoirieane-type bromo­diterpenoid isolated from *Laurencia yonaguniensis* Masuda et Abe, species inedita. The absolute stereochemistry was established as (1*S*,4*R*,5*R*,7*R*,10*S*,11*S*,14*R*). The structure displays inter- and intra­molecular O—H⋯O hydrogen bonding.

## Related literature

For background to neoirieane-type structures, see: Suzuki *et al.* (2002 ▶); Takahashi *et al.* (2002 ▶). For the related absolute configuration, see: Takahashi *et al.* (2007 ▶).
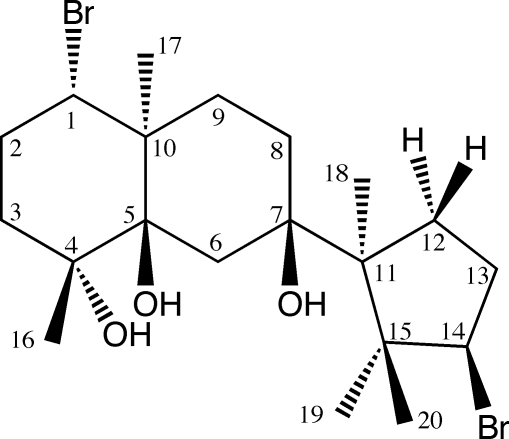


## Experimental

### 

#### Crystal data

C_20_H_34_Br_2_O_3_
*M* *_r_* = 482.29
Monoclinic, 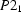

*a* = 7.5026 (2) Å
*b* = 11.3985 (3) Å
*c* = 12.1498 (5) Å
β = 94.9780 (3)°
*V* = 1035.11 (6) Å^3^
*Z* = 2
Mo *K*α radiation
μ = 3.94 mm^−1^
*T* = 296 K
0.30 × 0.20 × 0.20 mm

#### Data collection

Nonius KappaCCD diffractometer
Absorption correction: multi-scan (*DENZO-SMN*; Otwinowski & Minor, 1997 ▶) *T*_min_ = 0.402, *T*_max_ = 0.454
43586 measured reflections
6129 independent reflections
4774 reflections with *F* ^2^ > 2σ(*F* ^2^)
*R*_int_ = 0.110

#### Refinement

*R*[*F* ^2^ > 2σ(*F* ^2^)] = 0.062
*wR*(*F* ^2^) = 0.151
*S* = 1.14
6129 reflections
227 parameters
All H-atom parameters refined
Δρ_max_ = 0.66 e Å^−3^
Δρ_min_ = −0.44 e Å^−3^
Absolute structure: Flack (1983 ▶)
Flack parameter: −0.014 (12)

### 

Data collection: *KappaCCD Server Software* (Nonius, 1998 ▶); cell refinement: *DENZO-SMN* (Otwinowski & Minor, 1997 ▶); data reduction: *CrystalStructure* (Rigaku, 2007 ▶); program(s) used to solve structure: *SIR97* (Altomare *et al.*, 1999 ▶); program(s) used to refine structure: *SHELXL97* (Sheldrick, 2008 ▶); molecular graphics: *ORTEP-3* (Farrugia, 1997 ▶); software used to prepare material for publication: *publCIF* (Westrip, 2010 ▶).

## Supplementary Material

Crystal structure: contains datablocks global, I. DOI: 10.1107/S1600536810022336/fj2316sup1.cif

Structure factors: contains datablocks I. DOI: 10.1107/S1600536810022336/fj2316Isup2.hkl

Additional supplementary materials:  crystallographic information; 3D view; checkCIF report

## Figures and Tables

**Table 1:** Hydrogen-bond geometry (Å, °)

*D*—H⋯*A*	*D*—H	H⋯*A*	*D*⋯*A*	*D*—H⋯*A*
O1—H32⋯O3^i^	0.82	2.02	2.797 (4)	158
O3—H34⋯O2	0.82	1.96	2.691 (4)	148

Symmetry code: (i) 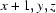.

**Fig. 1.**
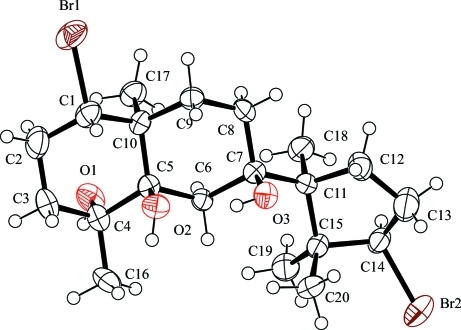
The structure of the title compound with ellipsoids at the 50% probability level and the atom numbering scheme.

**Fig. 2.**
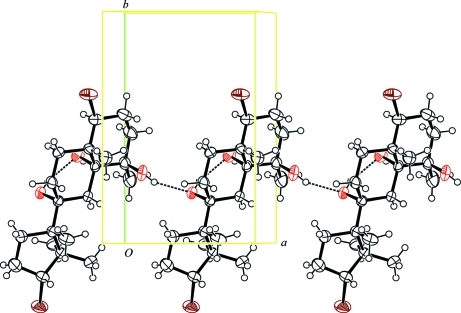
The packing diagram of the title compound. Inter and intramolecular hydrogen bonds are shown as dashed line.

## supplementary crystallographic information

### Comment

As part of our continuing chemotaxonomical studies on Japanese species of the red algal genus *Laurencia* (Rhodomelaceae, Ceramiales), we reported previously the structure of neoirietetraol (Takahashi *et al.*, 2002, Takahashi *et al.*, 2007), including the relative configuration and X-ray crystal structure, isolated from *Laurencia yonaguniensis *Masuda et Abe, species inedita (Masuda, *M*.; unpublished results), which was collected at Yonaguni Island, Okinawa, Japan. Further investigation of the related metabolites from this alga has led to the isolation of a new bromoditerpene, named neoirietriol, having a molecular formula of C_20_H_34_Br_2_O_3_, which was established by FD-LRMS (m/*z* 466, 464, 462 (1:2:1); M–H_2_O) and FAB-HRMS (m/*z* 479.0813; calcd for C_20_H_33_^79^Br_2_O_3_, 479.0796; M–H).

During the course of refinement of the structure, the Flack parameter converged to a value of -0.014 (12) within the derived limits as required for the correct enantiomorph of the structure. The absolute configuration of the title compound was established as (1*S*, 4*R*, 5*R*, 7*S*, 10*R*, 11*S*, 14*R*) (Fig. 1).

In the crystal, an intramolecular hydrogen bond was observed between O3···O2[distance 2.691 (4) Å] and an intermolecular hydrogen bond between O1···O3 (*x* + 1, *y*, *z*; distance 2.797 (4) Å) forming an infinite chain structure along the *a* axis (Fig. 2).

### Experimental

Isolation

The partially dried alga (40 g) was soaked in MeOH for 3 days. The MeOH solution was concentrated in *vacuo* and partitioned between Et_2_O and H_2_O. The Et_2_O solution was washed with water, dried over anhydrous Na_2_SO_4_, and evaporated to leave a dark-green oil (523 mg). The extract was fractionated by column chromatography on Si gel with a step gradient (hexane and ethyl acetate). The fraction (144 mg) eluted with hexane-EtOAc (3:1) was further subjected to preparative TLC with toluene-EtOAc (4:1) gave neoiretriol (40.8 mg, 7.8% based on the weight of MeOH extract).

Neoirietriol: mp 132–133 *^o^*C (from CH_2_Cl_2_/hexane (2:1)); [*a*]_D_^28^ -61¯ (c 0.53; CHCl_3_); ^1^H NMR (400 MHz; C_6_D_6_), d 0.28 (1*H*, br s, OH: D_2_O exchangeable), 0.50 (3*H*, s, H_3_-18), 0.54 (1*H*, m, Ha-8), 0.60 (1*H*, ddd, *J* = 13.2, 10.3, 5.4 Hz, Ha-12), 0.75 (1*H*, d, *J* = 2.4 Hz, OH: D_2_O exchangeable), 0.93 (1*H*, ddd, *J* = 13.7, 4.9, 2.4 Hz, Ha-3), 0.97 (3*H*, s, H_3_-20), 1.20 (3*H*, s, H_3_-19), 1.38 (3*H*, s, H_3_-17), 1.21 (1*H*, ddd, *J* = 13.2, 13.2, 4.4 Hz, Hb-12), 1.56 (1*H*, m, Ha-9), 1.67 (1*H*, ddd, *J* = 13.2, 13.2, 3.9 Hz, Hb-8), 1.76 (1*H*, dd, *J* = 14.2, 2.4 Hz, Ha-6), 1.83 (1*H*, m, Hb-9), 1.86 (1*H*, m, Ha-13), 1.95 (1*H*, ddd, ddd, *J* = 13.7, 9.3, 4.9 Hz, Hb-13), 2.03 (1*H*, m, Ha-2), 2.13 (1*H*, ddd, *J* = 13.7, 13.7, 4.9 Hz, Hb-3), 2.07 (1*H*, dd, *J *= 14.2, 2.4 Hz, Hb-6), 2.48 (1*H*, dddd, *J *= 13.8, 13.2, 12.7, 4.4 Hz, Hb-2), 4.01 (1*H*, dd, *J* = 10.3, 8.8 Hz, H14), 4.88 (1*H*, dd, *J* = 12.7, 4.4 Hz, H-1), 5.20 (1*H*, s, OH: D_2_O exchangeable); ^13^C NMR (100 MHz, DEPT; C_6_D_6_) d 18.8 (C, C17), 23.4 (CH_3_, C18), 23.5 (CH_3_, C19), 23.7 (CH_3_, C20), 26.7 (CH_3_, C16), 30.3 (CH_2_*x* 2, C8 and C12), 31.4 (CH_2_, C2), 32.2 (C, C9), 31.6 (CH_2_, C13), 31.7 (CH_2_, C6), 32.2 (CH_2_, C2), 38.3 (CH_2_, C3), 43.7 (C, C10), 48.5 (C, C15), 51.8 (C, C11), 65.2 (CH, C14), 65.7 (CH, C1), 75.3 (C, C4), 78.5 (C, C5), 81.7 (C, C7).

### Refinement

Refinement was performed using all reflections. The weighted *R*-factor (*wR*) and goodness of fit (*S*) are based on *F*^2^. *R*-factor (gt) are based on *F*. The threshold expression of *F*^2^ > 2.0 σ(*F*^2^) is used only for calculating *R*-factor (gt). Non-H atoms were refined anisotropically. H atoms were treated as riding models.

### Crystal data

**Table:**

C_20_H_34_Br_2_O_3_	*F*(000) = 496.00
*M**_r_* = 482.29	*D*_x_ = 1.547 Mg m^−^^3^
Monoclinic, *P*2_1_	Mo *K*α radiation, λ = 0.71069 Å
Hall symbol: P 2yb	Cell parameters from 1225 reflections
*a* = 7.5026 (2) Å	θ = 1.8–28.1°
*b* = 11.3985 (3) Å	µ = 3.94 mm^−^^1^
*c* = 12.1498 (5) Å	*T* = 296 K
β = 94.9780 (3)°	Prism, colorless
*V* = 1035.11 (6) Å^3^	0.30 × 0.20 × 0.20 mm
*Z* = 2	

### Data collection

**Table:**

Nonius KappaCCD diffractometer	6129 independent reflections
Radiation source: Mo Kα	4774 reflections with *F*^2^ > 2σ(*F*^2^)
horizonally mounted graphite crystal	*R*_int_ = 0.110
Detector resolution: 9 pixels mm^-1^	θ_max_ = 30.5°
ω scans	*h* = −10→10
Absorption correction: multi-scan (*DENZO-SMN*; Otwinowski & Minor, 1997)	*k* = −16→16
*T*_min_ = 0.402, *T*_max_ = 0.454	*l* = −17→17
43586 measured reflections	

### Refinement

**Table:**

Refinement on *F*^2^	*w* = 1/[σ^2^(*F*_o_^2^) + (0.0612*P*)^2^ + 1.0272*P*] where *P* = (*F*_o_^2^ + 2*F*_c_^2^)/3
*R*[*F*^2^ > 2σ(*F*^2^)] = 0.062	(Δ/σ)_max_ < 0.001
*wR*(*F*^2^) = 0.151	Δρ_max_ = 0.66 e Å^−^^3^
*S* = 1.14	Δρ_min_ = −0.44 e Å^−^^3^
6129 reflections	Absolute structure: Flack (1983)
227 parameters	Flack parameter: −0.014 (12)
All H-atom parameters refined	

### Special details

**Table:**

Refinement. Refinement was performed using all reflections. The weighted *R*-factor (*wR*) and goodness of fit (*S*) are based on *F*^2^. *R*-factor (gt) are based on *F*. The threshold expression of *F*^2^ > 2.0 σ (*F*^2^) is used only for calculating *R*-factor (gt).

### Fractional atomic coordinates and isotropic or equivalent isotropic displacement parameters (Å^2^)

**Table:**

	*x*	*y*	*z*	*U*_iso_*/*U*_eq_	
Br1	0.82169 (9)	0.64485 (5)	0.41310 (5)	0.05902 (18)	
Br2	0.45713 (9)	−0.27536 (5)	0.13400 (6)	0.05993 (18)	
O1	1.1510 (4)	0.3045 (3)	0.2533 (3)	0.0471 (8)	
O2	0.7088 (4)	0.3800 (3)	0.1271 (2)	0.0381 (7)	
O3	0.4700 (3)	0.2183 (3)	0.1812 (2)	0.0357 (6)	
C1	0.8500 (7)	0.5365 (4)	0.2880 (4)	0.0421 (10)	
C2	1.0332 (8)	0.5566 (5)	0.2479 (5)	0.0552 (14)	
C3	1.0585 (8)	0.4788 (5)	0.1477 (5)	0.0522 (13)	
C4	1.0244 (6)	0.3474 (4)	0.1691 (4)	0.0378 (9)	
C5	0.8363 (5)	0.3340 (3)	0.2131 (3)	0.0299 (8)	
C6	0.7899 (5)	0.2032 (3)	0.2322 (3)	0.0286 (8)	
C7	0.6015 (5)	0.1830 (3)	0.2705 (3)	0.0298 (8)	
C8	0.5726 (6)	0.2635 (4)	0.3683 (4)	0.0381 (10)	
C9	0.6148 (6)	0.3931 (4)	0.3459 (4)	0.0355 (9)	
C10	0.8119 (6)	0.4086 (3)	0.3189 (3)	0.0316 (8)	
C11	0.5662 (6)	0.0505 (3)	0.2964 (3)	0.0310 (8)	
C12	0.3623 (7)	0.0296 (4)	0.3094 (5)	0.0463 (11)	
C13	0.3165 (9)	−0.0954 (5)	0.2707 (7)	0.0631 (17)	
C14	0.4932 (7)	−0.1454 (4)	0.2415 (4)	0.0395 (10)	
C15	0.6091 (6)	−0.0428 (4)	0.2044 (4)	0.0338 (9)	
C16	1.0421 (8)	0.2773 (6)	0.0629 (4)	0.0538 (14)	
C17	0.9349 (6)	0.3707 (4)	0.4209 (4)	0.0404 (10)	
C18	0.6753 (8)	0.0176 (4)	0.4068 (4)	0.0445 (11)	
C19	0.8045 (7)	−0.0813 (4)	0.2069 (5)	0.0460 (11)	
C20	0.5443 (7)	−0.0058 (4)	0.0862 (4)	0.0419 (11)	
H1	0.7610	0.5588	0.2279	0.051*	
H2	1.1248	0.5387	0.3068	0.066*	
H3	1.0455	0.6384	0.2278	0.066*	
H4	1.1797	0.4883	0.1271	0.063*	
H5	0.9775	0.5048	0.0860	0.063*	
H6	0.7994	0.1604	0.1639	0.034*	
H7	0.8777	0.1708	0.2872	0.034*	
H8	0.6481	0.2370	0.4324	0.046*	
H9	0.4491	0.2571	0.3856	0.046*	
H10	0.5939	0.4397	0.4103	0.043*	
H11	0.5354	0.4213	0.2843	0.043*	
H12	0.3382	0.0392	0.3860	0.056*	
H13	0.2903	0.0857	0.2651	0.056*	
H14	0.2689	−0.1406	0.3291	0.076*	
H15	0.2296	−0.0948	0.2068	0.076*	
H16	0.5541	−0.1780	0.3094	0.047*	
H17	1.1539	0.2958	0.0341	0.065*	
H18	0.9453	0.2971	0.0092	0.065*	
H19	1.0381	0.1949	0.0789	0.065*	
H20	1.0525	0.4023	0.4159	0.048*	
H21	0.9414	0.2866	0.4236	0.048*	
H22	0.8874	0.3994	0.4866	0.048*	
H23	0.7981	0.0396	0.4033	0.053*	
H24	0.6679	−0.0655	0.4186	0.053*	
H25	0.6272	0.0583	0.4668	0.053*	
H26	0.8741	−0.0195	0.1783	0.055*	
H27	0.8129	−0.1503	0.1623	0.055*	
H28	0.8493	−0.0983	0.2816	0.055*	
H29	0.4186	0.0112	0.0822	0.050*	
H30	0.5654	−0.0684	0.0360	0.050*	
H31	0.6084	0.0629	0.0664	0.050*	
H32	1.2265	0.2650	0.2253	0.057*	
H33	0.7350	0.3567	0.0667	0.046*	
H34	0.5132	0.2691	0.1438	0.043*	

### Atomic displacement parameters (Å^2^)

**Table:**

	*U*^11^	*U*^22^	*U*^33^	*U*^12^	*U*^13^	*U*^23^
Br1	0.0831 (4)	0.0325 (2)	0.0605 (3)	−0.0029 (2)	0.0009 (2)	−0.0086 (2)
Br2	0.0686 (3)	0.0386 (2)	0.0705 (3)	−0.0076 (2)	−0.0061 (2)	−0.0147 (2)
O1	0.0255 (16)	0.063 (2)	0.053 (2)	0.0083 (15)	0.0065 (14)	0.0017 (18)
O2	0.0400 (17)	0.0439 (18)	0.0292 (14)	0.0058 (14)	−0.0033 (12)	0.0043 (13)
O3	0.0261 (13)	0.0377 (15)	0.0425 (15)	0.0037 (13)	−0.0013 (11)	0.0025 (15)
C1	0.050 (2)	0.032 (2)	0.044 (2)	0.000 (2)	0.000 (2)	0.0004 (19)
C2	0.058 (3)	0.036 (2)	0.073 (3)	−0.013 (2)	0.012 (2)	0.001 (2)
C3	0.050 (3)	0.052 (3)	0.056 (3)	−0.012 (2)	0.017 (2)	0.014 (2)
C4	0.032 (2)	0.041 (2)	0.041 (2)	−0.0017 (18)	0.0067 (18)	0.0063 (19)
C5	0.0233 (19)	0.034 (2)	0.032 (2)	0.0022 (16)	−0.0020 (15)	0.0026 (16)
C6	0.0237 (18)	0.029 (2)	0.0336 (19)	0.0022 (14)	0.0040 (14)	−0.0009 (15)
C7	0.027 (2)	0.0292 (17)	0.0326 (19)	0.0031 (16)	0.0006 (17)	−0.0006 (15)
C8	0.035 (2)	0.040 (2)	0.042 (2)	−0.0044 (18)	0.0175 (19)	−0.0039 (19)
C9	0.036 (2)	0.031 (2)	0.041 (2)	0.0022 (18)	0.0081 (19)	−0.0057 (18)
C10	0.031 (2)	0.0289 (19)	0.034 (2)	−0.0022 (16)	−0.0024 (16)	−0.0005 (16)
C11	0.033 (2)	0.031 (2)	0.0299 (19)	−0.0013 (17)	0.0071 (16)	−0.0020 (16)
C12	0.041 (2)	0.038 (2)	0.062 (3)	−0.001 (2)	0.017 (2)	−0.001 (2)
C13	0.047 (3)	0.048 (3)	0.097 (5)	−0.008 (2)	0.018 (3)	−0.014 (3)
C14	0.044 (2)	0.032 (2)	0.042 (2)	−0.0083 (19)	0.001 (2)	−0.0053 (18)
C15	0.030 (2)	0.030 (2)	0.041 (2)	−0.0017 (17)	0.0012 (18)	−0.0043 (17)
C16	0.047 (3)	0.072 (3)	0.046 (2)	−0.004 (2)	0.023 (2)	−0.005 (2)
C17	0.042 (2)	0.043 (2)	0.035 (2)	−0.002 (2)	−0.0061 (18)	−0.0006 (19)
C18	0.065 (3)	0.037 (2)	0.031 (2)	−0.008 (2)	0.000 (2)	0.0044 (19)
C19	0.044 (2)	0.036 (2)	0.058 (3)	0.006 (2)	0.009 (2)	−0.003 (2)
C20	0.052 (3)	0.037 (2)	0.036 (2)	0.001 (2)	0.000 (2)	−0.0084 (19)

### Geometric parameters (Å, °)

**Table:**

Br1—C1	1.984 (5)	C2—H3	0.970
Br2—C14	1.978 (4)	C3—H4	0.970
O1—C4	1.421 (6)	C3—H5	0.970
O2—C5	1.452 (5)	C6—H6	0.970
O3—C7	1.458 (5)	C6—H7	0.970
C1—C2	1.515 (9)	C8—H8	0.970
C1—C10	1.539 (6)	C8—H9	0.970
C2—C3	1.531 (9)	C9—H10	0.970
C3—C4	1.545 (8)	C9—H11	0.970
C4—C5	1.559 (6)	C12—H12	0.970
C4—C16	1.533 (8)	C12—H13	0.970
C5—C6	1.553 (5)	C13—H14	0.970
C5—C10	1.565 (6)	C13—H15	0.970
C6—C7	1.543 (6)	C14—H16	0.980
C7—C8	1.532 (6)	C16—H17	0.960
C7—C11	1.570 (6)	C16—H18	0.960
C8—C9	1.541 (6)	C16—H19	0.960
C9—C10	1.552 (6)	C17—H20	0.960
C10—C17	1.541 (6)	C17—H21	0.960
C11—C12	1.569 (7)	C17—H22	0.960
C11—C15	1.597 (6)	C18—H23	0.960
C11—C18	1.556 (6)	C18—H24	0.960
C12—C13	1.530 (8)	C18—H25	0.960
C13—C14	1.513 (8)	C19—H26	0.960
C14—C15	1.547 (6)	C19—H27	0.960
C15—C19	1.528 (7)	C19—H28	0.960
C15—C20	1.534 (6)	C20—H29	0.960
O1—H32	0.820	C20—H30	0.960
C1—H1	0.980	C20—H31	0.960
C2—H2	0.970		
			
O1···O3^i^	2.797 (4)	H15···H27^iii^	3.189
O1···C8^i^	3.378 (5)	H15···H28^iii^	3.069
O1···C12^i^	3.551 (6)	H15···H33^iv^	3.401
O2···C20^ii^	3.341 (5)	H16···Br1^viii^	3.043
O3···O1^iii^	2.797 (4)	H16···H1^viii^	3.558
O3···C16^iii^	3.468 (6)	H17···O3^i^	2.977
C8···O1^iii^	3.378 (5)	H17···C19^xii^	3.285
C12···O1^iii^	3.551 (6)	H17···C20^xii^	3.599
C16···O3^i^	3.468 (6)	H17···H26^xii^	3.323
C20···O2^iv^	3.341 (6)	H17···H27^xii^	2.498
Br1···H9^v^	3.551	H17···H30^xii^	2.805
Br1···H12^v^	3.058	H17···H33	3.276
Br1···H16^vi^	3.043	H17···H34^i^	2.917
Br1···H21^vii^	3.015	H18)···Br2^ii^	3.452
Br1···H24^vi^	3.500	H18)···H3^xi^	3.407
Br1···H28^vi^	3.350	H18···H5^xi^	3.591
Br2···H1^viii^	3.101	H18···H15^ii^	3.087
Br2···H3^ix^	3.523	H18···H26^xii^	3.454
Br2···H4^ix^	3.401	H18···H27^xii^	2.941
Br2···H18^iv^	3.452	H18···H33	1.905
Br2···H31^iv^	3.060	H19···O3^i^	3.379
Br2···H33^iv^	3.111	H19···H5^xi^	2.946
Br2···H34^iv^	3.439	H19···H13^i^	3.083
O1···H9)^i^	2.694	H19···H29^i^	3.538
O1···H11^i^	3.169	H19···H33	2.922
O1···H13^i^	2.703	H20···C18^vii^	3.126
O1···H34^i^	3.153	H20···H9^i^	3.453
O2···H29^ii^	3.032	H20···H23^vii^	2.847
O2···H30^ii^	2.792	H20···H24^vii^	2.802
O2···H33	0.820	H20···H25^vii^	3.221
O2···H34	1.960	H21···Br1^xiii^	3.015
O3···H17^iii^	2.977	H22···C12^v^	3.558
O3···H19^iii^	3.379	H22···C13^v^	3.438
O3···H30^ii^	3.581	H22···H12^v^	2.873
O3···H32^iii^	2.020	H22···H14^v^	2.656
O3···H33	2.976	H22···H23^vii^	3.060
O3···H34	0.820	H22···H24^vii^	3.458
C1···H33	3.432	H22···H28^vii^	3.297
C3···H33	2.895	H23···C17^xiii^	3.398
C4···H33	2.410	H23···H2^xiii^	3.520
C5···H33	1.891	H23···H20^xiii^	2.847
C5···H34	2.604	H23···H22^xiii^	3.060
C6···H33	2.671	H24···Br1^viii^	3.500
C6···H34	2.376	H24···C17^xiii^	3.494
C7···H32^iii^	2.970	H24···H9^x^	3.299
C7···H33	3.389	H24···H10^x^	2.982
C7···H34	1.896	H24···H20^xiii^	2.802
C8···H32^iii^	2.996	H24···H22^xiii^	3.458
C8···H34	2.727	H25···C9^x^	3.569
C9···H25^v^	3.569	H25···H2^xiii^	3.193
C9···H32^iii^	3.468	H25···H10^x^	2.690
C9···H34	2.878	H25···H20^xiii^	3.221
C10···H33	3.125	H26···C13^i^	3.518
C10···H34	3.354	H26···H5^xi^	3.500
C11···H32^iii^	3.582	H26···H13^i^	3.424
C11···H34	3.111	H26···H15^i^	2.794
C12···H10^x^	3.544	H26···H17^xi^	3.323
C12···H22^x^	3.558	H26···H18^xi^	3.454
C12···H32^iii^	3.017	H27···C16^xi^	3.142
C13···H22^x^	3.438	H27···H1^viii^	3.440
C13···H26^iii^	3.518	H27···H3^viii^	3.039
C13···H28^iii^	3.519	H27···H15^i^	3.189
C16···H5^xi^	3.591	H27···H17^xi^	2.498
C16···H27^xii^	3.142	H27···H18^xi^	2.941
C16···H33	2.480	H28···Br1^viii^	3.350
C16···H34^i^	3.587	H28···C13^i^	3.519
C17···H14^v^	3.520	H28···H3^viii^	3.431
C17···H23^vii^	3.398	H28···H14^i^	3.188
C17···H24^vii^	3.494	H28···H15^i^	3.069
C18···H10^x^	3.253	H28···H22^xiii^	3.297
C18···H20^xiii^	3.126	H29···O2^iv^	3.032
C19···H15^i^	3.193	H29···H5^iv^	3.458
C19···H17^xi^	3.285	H29···H19^iii^	3.538
C20···H4^xi^	3.455	H29···H33^iv^	2.709
C20···H17^xi^	3.599	H29···H34	3.101
C20···H33^iv^	3.101	H30···O2^iv^	2.792
C20···H34	3.224	H30···O3^iv^	3.581
H1···Br2^vi^	3.101	H30···H4^xi^	2.943
H1···H16^vi^	3.558	H30···H17^xi^	2.805
H1···H27^vi^	3.440	H30···H33^iv^	2.622
H1···H33	3.019	H30···H34^iv^	2.886
H2···H11^i^	3.391	H31···Br2^ii^	3.060
H2···H23^vii^	3.520	H31···H4^xi^	3.070
H2···H25^vii^	3.193	H31···H33	3.482
H3···Br2^xiv^	3.523	H31···H34	2.653
H3···H14^xiv^	3.212	H32···O3^i^	2.020
H3···H15^xiv^	3.359	H32···C7^i^	2.970
H3···H18^xii^	3.407	H32···C8^i^	2.996
H3···H27^vi^	3.039	H32···C9^i^	3.468
H3···H28^vi^	3.431	H32···C11^i^	3.582
H4···Br2^xiv^	3.401	H32···C12^i^	3.017
H4···C20^xii^	3.455	H32···H9^i^	2.454
H4···H11^i^	3.235	H32···H11^i^	2.961
H4···H30^xii^	2.943	H32···H12^i^	3.295
H4···H31^xii^	3.070	H32···H13^i^	2.145
H4···H34^i^	3.528	H32···H34^i^	2.445
H5···C16^xii^	3.591	H33···Br2^ii^	3.111
H5···H18^xii^	3.591	H33···O2	0.820
H5···H19^xii^	2.946	H33···O3	2.976
H5···H26^xii^	3.500	H33···C1	3.432
H5···H29^ii^	3.458	H33···C3	2.895
H5···H33	2.478	H33···C4	2.410
H6···H33	2.557	H33···C5	1.891
H6···H34	2.473	H33···C6	2.671
H7···H13^i^	3.277	H33···C7	3.389
H7···H33	3.510	H33···C10	3.125
H7···H34	3.309	H33···C16	2.480
H8···H14^v^	3.227	H33···C20^ii^	3.101
H8···H34	3.583	H33···H1	3.019
H9···Br1^x^	3.551	H33···H5	2.478
H9···O1^iii^	2.694	H33···H6	2.557
H9···H20^iii^	3.453	H33···H7	3.510
H9···H24^v^	3.299	H33···H11	3.235
H9···H32^iii^	2.454	H33···H15^ii^	3.401
H9···H34	3.021	H33···H17	3.276
H10···C12^v^	3.544	H33···H18	1.905
H10···C18^v^	3.253	H33···H19	2.922
H10···H12^v^	2.729	H33···H29^ii^	2.709
H10···H14^v^	3.370	H33···H30^ii^	2.622
H10···H24^v^	2.982	H33···H31	3.482
H10···H25^v^	2.690	H33···H34	2.217
H11···O1^iii^	3.169	H34···Br2^ii^	3.439
H11···H2^iii^	3.391	H34···O1^iii^	3.153
H11···H4^iii^	3.235	H34···O2	1.960
H11···H32^iii^	2.961	H34···O3	0.820
H11···H33	3.235	H34···C5	2.604
H11···H34	2.430	H34···C6	2.376
H12···Br1^x^	3.058	H34···C7	1.896
H12···H10^x^	2.729	H34···C8	2.727
H12···H22^x^	2.873	H34···C9	2.878
H12···H32^iii^	3.295	H34···C10	3.354
H13···O1^iii^	2.703	H34···C11	3.111
H13···H7^iii^	3.277	H34···C16^iii^	3.587
H13···H19^iii^	3.083	H34···C20	3.224
H13···H26^iii^	3.424	H34···H4^iii^	3.528
H13···H32^iii^	2.145	H34···H6	2.473
H13···H34	3.125	H34···H7	3.309
H14···C17^x^	3.520	H34···H8	3.583
H14···H3^ix^	3.212	H34···H9	3.021
H14···H8^x^	3.227	H34···H11	2.430
H14···H10^x^	3.370	H34···H13	3.125
H14···H22^x^	2.656	H34···H17^iii^	2.917
H14···H28^iii^	3.188	H34···H29	3.101
H15···C19^iii^	3.193	H34···H30^ii^	2.886
H15···H3^ix^	3.359	H34···H31	2.653
H15···H18^iv^	3.087	H34···H32^iii^	2.445
H15···H26^iii^	2.794	H34···H33	2.217
			
Br1—C1—C2	108.2 (3)	C4—C3—H4	108.9
Br1—C1—C10	111.7 (3)	C4—C3—H5	108.9
C2—C1—C10	114.5 (4)	H4—C3—H5	107.8
C1—C2—C3	110.3 (4)	C5—C6—H6	108.7
C2—C3—C4	113.2 (5)	C5—C6—H7	108.7
O1—C4—C3	110.2 (4)	C7—C6—H6	108.7
O1—C4—C5	106.7 (3)	C7—C6—H7	108.7
O1—C4—C16	109.0 (4)	H6—C6—H7	107.6
C3—C4—C5	108.7 (4)	C7—C8—H8	109.0
C3—C4—C16	109.7 (4)	C7—C8—H9	109.0
C5—C4—C16	112.6 (4)	C9—C8—H8	109.0
O2—C5—C4	106.1 (3)	C9—C8—H9	109.0
O2—C5—C6	108.2 (3)	H8—C8—H9	107.8
O2—C5—C10	106.2 (3)	C8—C9—H10	109.4
C4—C5—C6	111.5 (3)	C8—C9—H11	109.4
C4—C5—C10	113.7 (3)	C10—C9—H10	109.4
C6—C5—C10	110.8 (3)	C10—C9—H11	109.4
C5—C6—C7	114.3 (3)	H10—C9—H11	108.0
O3—C7—C6	108.2 (3)	C11—C12—H12	110.1
O3—C7—C8	106.3 (3)	C11—C12—H13	110.1
O3—C7—C11	107.4 (3)	C13—C12—H12	110.1
C6—C7—C8	109.9 (3)	C13—C12—H13	110.1
C6—C7—C11	112.3 (3)	H12—C12—H13	108.5
C8—C7—C11	112.5 (3)	C12—C13—H14	110.9
C7—C8—C9	113.0 (4)	C12—C13—H15	110.9
C8—C9—C10	111.1 (3)	C14—C13—H14	110.9
C1—C10—C5	106.1 (3)	C14—C13—H15	110.9
C1—C10—C9	111.1 (3)	H14—C13—H15	108.9
C1—C10—C17	110.5 (3)	Br2—C14—H16	107.4
C5—C10—C9	107.1 (3)	C13—C14—H16	107.4
C5—C10—C17	113.8 (3)	C15—C14—H16	107.4
C9—C10—C17	108.3 (3)	C4—C16—H17	109.5
C7—C11—C12	110.5 (3)	C4—C16—H18	109.5
C7—C11—C15	116.9 (3)	C4—C16—H19	109.5
C7—C11—C18	108.6 (3)	H17—C16—H18	109.5
C12—C11—C15	103.1 (3)	H17—C16—H19	109.5
C12—C11—C18	108.7 (4)	H18—C16—H19	109.5
C15—C11—C18	108.7 (3)	C10—C17—H20	109.5
C11—C12—C13	107.8 (4)	C10—C17—H21	109.5
C12—C13—C14	104.2 (4)	C10—C17—H22	109.5
Br2—C14—C13	111.4 (3)	H20—C17—H21	109.5
Br2—C14—C15	114.9 (3)	H20—C17—H22	109.5
C13—C14—C15	108.0 (4)	H21—C17—H22	109.5
C11—C15—C14	98.4 (3)	C11—C18—H23	109.5
C11—C15—C19	115.4 (3)	C11—C18—H24	109.5
C11—C15—C20	113.9 (3)	C11—C18—H25	109.5
C14—C15—C19	109.9 (4)	H23—C18—H24	109.5
C14—C15—C20	109.9 (3)	H23—C18—H25	109.5
C19—C15—C20	108.9 (4)	H24—C18—H25	109.5
C4—O1—H32	109.5	C15—C19—H26	109.5
C5—O2—H33	109.5	C15—C19—H27	109.5
C7—O3—H34	109.5	C15—C19—H28	109.5
Br1—C1—H1	107.4	H26—C19—H27	109.5
C2—C1—H1	107.4	H26—C19—H28	109.5
C10—C1—H1	107.4	H27—C19—H28	109.5
C1—C2—H2	109.6	C15—C20—H29	109.5
C1—C2—H3	109.6	C15—C20—H30	109.5
C3—C2—H2	109.6	C15—C20—H31	109.5
C3—C2—H3	109.6	H29—C20—H30	109.5
H2—C2—H3	108.1	H29—C20—H31	109.5
C2—C3—H4	108.9	H30—C20—H31	109.5
C2—C3—H5	108.9		

Symmetry codes: (i) *x*+1, *y*, *z*; (ii) −*x*+1, *y*+1/2, −*z*; (iii) *x*−1, *y*, *z*; (iv) −*x*+1, *y*−1/2, −*z*; (v) −*x*+1, *y*+1/2, −*z*+1; (vi) *x*, *y*+1, *z*; (vii) −*x*+2, *y*+1/2, −*z*+1; (viii) *x*, *y*−1, *z*; (ix) *x*−1, *y*−1, *z*; (x) −*x*+1, *y*−1/2, −*z*+1; (xi) −*x*+2, *y*−1/2, −*z*; (xii) −*x*+2, *y*+1/2, −*z*; (xiii) −*x*+2, *y*−1/2, −*z*+1; (xiv) *x*+1, *y*+1, *z*.

### Hydrogen-bond geometry (Å, °)

**Table:**

*D*—H···*A*	*D*—H	H···*A*	*D*···*A*	*D*—H···*A*
O1—H32···O3^i^	0.82	2.02	2.797 (4)	158
O3—H34···O2	0.82	1.96	2.691 (4)	148

Symmetry codes: (i) *x*+1, *y*, *z*.
